# CXCL16 Stimulates Antigen-Induced MAIT Cell Accumulation but Trafficking During Lung Infection Is CXCR6-Independent

**DOI:** 10.3389/fimmu.2020.01773

**Published:** 2020-08-07

**Authors:** Huifeng Yu, Amy Yang, Ligong Liu, Jeffrey Y. W. Mak, David P. Fairlie, Siobhan Cowley

**Affiliations:** ^1^Laboratory of Mucosal Pathogens and Cellular Immunology, Division of Bacterial Parasitic and Allergenic Products, Center for Biologics Evaluation and Research, U.S. Food and Drug Administration, Silver Spring, MD, United States; ^2^Institute for Molecular Bioscience, The University of Queensland, Brisbane, QLD, Australia; ^3^Australian Research Council Centre of Excellence in Advanced Molecular Imaging, The University of Queensland, Brisbane, QLD, Australia

**Keywords:** MAIT cells, pulmonary infection, *F. tularensis*, CXCR6, CXCL16

## Abstract

Mucosa-associated invariant T (MAIT) cells are a unique T cell subset that contributes to protective immunity against microbial pathogens, but little is known about the role of chemokines in recruiting MAIT cells to the site of infection. Pulmonary infection with *Francisella tularensis* live vaccine strain (LVS) stimulates the accrual of large numbers of MAIT cells in the lungs of mice. Using this infection model, we find that MAIT cells are predominantly CXCR6^+^ but do not require CXCR6 for accumulation in the lungs. However, CXCR6 does contribute to long-term retention of MAIT cells in the airway lumen after clearance of the infection. We also find that MAIT cells are not recruited from secondary lymphoid organs and largely proliferate *in situ* in the lungs after infection. Nevertheless, the only known ligand for CXCR6, CXCL16, is sufficient to drive MAIT cell accumulation in the lungs in the absence of infection when administered in combination with the MAIT cell antigen 5-OP-RU. Overall, this new data advances the understanding of mechanisms that facilitate MAIT cell accumulation and retention in the lungs.

## Introduction

Mucosa-associated invariant T (MAIT) cells are innate-like T cells that play important roles in protective immunity against microbial infections (1–5). MAIT cells are activated by riboflavin-related metabolites presented by the major histocompatibility complex (MHC) class I related protein (MR1). Riboflavin-related metabolites such as 5-(2-oxopropylideneamino)-6-d-ribitylaminouracil (5-OP-RU) are present in many bacteria, yeast, and plants, but not in mammals and other animals (6–8). Several pathogens such as *Francisella tularensis* live vaccine strain (LVS), *Legionella longbeachae*, and *Salmonella typhimurium* BRD509 have been reported to activate MAIT cells *in vivo* (9–11). MAIT cells have been implicated in anti-tumor responses and the exacerbation/amelioration of autoimmune diseases, including diabetes I, multiple sclerosis and gut-associated diseases like colitis (12–14).

Chemokines are differentially expressed in different tissues and inflammatory microenvironments and guide the homing of specific leukocyte subsets via interactions with differential cell surface chemokine receptors (15–17). Chemokines play critical roles in immunity and inflammation, including immune system development, leukocyte positioning, lymphocyte migration, and phagocyte activation (15, 18, 19). These important proteins and their receptors have been targeted as clinical therapies and investigated as biomarkers for certain human diseases (19).

In humans, MAIT cells have been observed in numerous tissues, including the lungs, intestinal lamina propria, liver, and peripheral blood (20–23). Human peripheral blood MAIT cells exhibited high levels of CCR6 and CXCR6, heterogenous levels of CXCR4, and intermediate expression of CCR9 under steady-state conditions, which may reflect the potential for circulating MAIT cells traffic to different tissues (21). Indeed, numerous reports have noted a significant decrease in the number of MAIT cells present in the peripheral blood of patients during infections, suggesting their recruitment from the blood to the site of infection. In contrast, MAIT cells in naïve pathogen-free wild type mice were present in low numbers in most tissues (24, 25), including the blood (0.09% of total αβT cells). Nevertheless, MAIT cells in the lungs of naïve mice were largely positive for CXCR6 and low for CCR9 (25).

Little is known about the role of chemokines and their receptors in regulating MAIT cell localization in tissues. It is unclear whether MAIT cells are recruited from peripheral tissues and local lymph nodes during inflammation, or whether they largely proliferate *in situ* at the site of infection. Since our previous work showed that MAIT cells robustly accumulated in the lungs of mice during pulmonary *F. tularensis* LVS infection, we used this model to investigate the role of proliferation and chemokine recruitment in MAIT cell expansion in the lungs after infection (1, 9). Here we find that, despite being predominantly CXCR6^+^, MAIT cells do not require CXCR6 for accumulation at the site of infection. We further find that MAIT cell accumulation is not driven by recruitment from secondary lymphoid organs, and that the majority of MAIT cells proliferate *in situ* in the lungs after pulmonary *F. tularensis* LVS infection. Surprisingly, however, the only known ligand for CXCR6, CXCL16, was sufficient to drive MAIT cell accumulation in the lungs of naïve mice when administered intranasally with the MAIT cell antigen 5-OP-RU (26).

## Materials and Methods

### Mice and Infection

*F. tularensis* LVS (ATCC) was grown and frozen as previously described (27). MR1 KO mice (28) and Vα19iTgCα^−/−^MR1^+/+^ transgenic mice exclusively expressing the canonical TCR Vα19-Jα33 of mouse MAIT cells (29) were obtained from Ted Hansen (Washington University in St. Louis, St. Louis, MO) and bred at CBER/FDA. Wild type mice (C57BL6J #000664) and CXCR6^−/−^mice (#005693) were purchased from The Jackson Laboratory. Animals were housed in a barrier environment at CBER/FDA, and procedures were performed according to approved protocols under the FDA Animal Care and Use Committee guidelines. Bacteria were diluted in PBS (Gibco, Life Technologies), and intranasal (IN) infections were performed by delivering 1 or 2 × 10^2^ LVS colony-forming units (CFU) in a volume of 25 μl to anesthetized mice.

For *in vivo* MAIT cell induction therapy, the first dose consisted of a combination of 30 μg Pam_2_CSK_4_ (Invivogen) and MAIT cell ligands (5-OP-RU or Ac-6-FP, 37.5 μl of an 8 μM solution) administrated IN per mouse. The second and third doses consisted of 5-OP-RU or Ac-6-FP (37.5 μl of a 4 μM solution) administrated IN per mouse. For MAIT cell induction therapies using chemokines, the relevant recombinant chemokines (CXCL16, heat-denatured CXCL16, and CCL24) were administered in the following regimen: the first dose consisted of a combination of 3 μg chemokine and MAIT cell ligands (5-OP-RU or Ac-6-FP, 37.5 μl of an 8 μM solution) administrated IN per mouse. The second and third doses consisted of 3 μg chemokine and 5-OP-RU or Ac-6-FP (37.5 μl of a 4 μM solution) administrated IN per mouse. CXCL16 and CCL24 were purchased from R&D systems. CXCL16 was heat-denatured by incubation at 100°C for 10 min. In all cases, lungs were harvested and processed for flow cytometry analyses 6 days after the first dose.

For FTY720 (Sigma) treatment, mice were injected daily i.p. with 0.5 mg/kg/day of FTY720 dissolved in sterile PBS after day 2 of LVS IN infection. Untreated control mice received an equivalent volume of sterile PBS. For BrdU treatment, mice were administered BrdU (62.5 μl of 10 mg/ml of BrdU) IN daily on days 6–9 after infection. BrdU staining was performed according to the manufacturer's instructions (BD).

### Preparation of Synthetic Ligands

Synthetic 5-OP-RU (as a DMSO solution) and Ac-6-FP were prepared according to previously published procedures (26, 30).

### Preparation of Single-Cell Suspensions

To prepare lung cells for flow cytometry, the lungs were excised and transferred to a Petri dish and chopped with a sharp scissors for ~30 s, or until no large pieces were visible. The pieces should be small enough to pass through 10 ml serological pipettes. The lung pieces were incubated with 3 ml of DMEM containing 10% FBS, collagenase D (Sigma, 0.5 mg/ml) for 1 h at 37°C in 5% CO_2_. Released cells were filtered through a 40-μm filter, subjected to ACK lysis, washed in PBS + 2% FBS, and passed through a 40-μm filter again. Live cells were enumerated using a hemocytometer after dilution in trypan blue. For the experiments shown in **Figures 3A,B**, the airway lumen cells and lung parenchyma cells were processed separately for each lung. For each mouse, the bronchoalveolar lavage (BAL) fluid was harvested by inserting a 18G catheter into an incision in the trachea and the airway lumen was washed four times with PBS. The resulting cells were processed for flow cytometry staining (airway lumen cells). After removal of the airway lumen cells, the lungs were then excised, and the tissue processed for flow cytometry as described above (lung parenchyma cells). For cells from draining LNs, thymus, and spleen, the organs were minced and the cell suspensions were passed through a 40-μm filter. Cells from the lamina propria were isolated according to a previous publication (31).

### *In vitro* Bone Marrow-Derived Macrophage Co-cultures

BMMØ were cultured as previously described. Briefly, bone marrow was flushed from femurs of healthy wild type mice with Dulbecco minimal essential medium (DMEM; Life Technologies) supplemented with 10% heat-inactivated fetal calf serum (FCS; HyClone), 10% L-929 conditioned medium, 0.2 mM L-glutamine (Life Technologies), 1 mM HEPES buffer (Life Technologies), and 0.1 mM non-essential amino acids (Life Technologies) [complete DMEM (cDMEM)]. Cells were washed, a single cell suspension prepared, and cells plated at 1 × 10^6^ in 48-well plates, or 2 × 10^6^ in 24-well plates, in cDMEM supplemented with 50 μg/ml gentamycin (Life Technologies) and incubated at 37°C in 5% CO_2_. After 1 day of incubation, the medium was replaced with antibiotic-free cDMEM, and the cells were incubated for an additional 6 days at 37°C in 5% CO_2_. The medium was replaced with fresh, gentamycin-free cDMEM every 2 days during the 7-day incubation. Vα19iTgCα^−/−^MR1^+/+^ mice were used as source of MAIT cells for co-culture with BMMØs. Total Thy1.2^+^ T cells were purified from Vα19iTgCα^−/−^MR1^+/+^ mouse spleens using a Thy1.2^+^ cell enrichment column (Life Technologies), according to the manufacturer's recommendations. MAIT cells were added to the wells at a ratio of 1 T cell to 2 macrophages. Recombinant CXCL16 (200 ng/ml) and 5-OP-RU (9.3 μM) were added to the cultures at the same time as the MAIT cells, and supernatants were collected for cytokine analyses (ThermoFisher Luminex assay for Cytokine & chemokine 26-Plex Mouse ProcartaPlex™ Panel 1) 16 h later.

### Flow Cytometry Analyses and Intracellular Cytokine Staining

Cells were stained for a panel of murine cell surface markers and analyzed by using a BD LSR Fortessa flow cytometer and FlowJo software. Ab clones used included GK1.5 (anti-CD4), 53-6.7 (anti-CD8α), H57-597 (anti-TCR β-chain), MP6-XT22 (anti-TNFα), TC1118H10.1 (anti-IL17A), XMG-1.2 (anti-IFN-γ), 2E7 (anti-CD103), M1/70 (anti-CD11b), 2G12 (anti-CCR4), HM-CCR5 (anti-CCR5), 29-2L17 (anti-CCR6), CXCR3-173 (anti-CXCR3), DATK32 (anti-α4β7), 2E7 (anti-CCR9), SA051D1 (anti-CXCR4), SA203G11 (anti-CCR2), J073E5 (anti-CCR3), CXCR3-173 (anti-CXCR3), 4B12 (anti-CCR7), SA051D1 (anti-CXCR6) were obtained from BioLegend. MR1 tetramers were obtained from the National Institutes of Health Tetramer Core Facility (Atlanta, GA). Live/Dead Near IR stain (Molecular Probes) was included in all staining protocols. Optimal antibody concentrations were determined in separate experiments, and appropriate fluorochrome-labeled isotype control antibodies or “fluorescence minus one” (FMO) controls were used throughout. In all cases, cells were first gated on singlets (forward scatter-width or forward scatter-area vs. -height) and live cells (Live/Dead Near IR negative) before further analyses.

To monitor the expression of TNF, IFN-γ, and IL-17A, lung cells were incubated in complete Dulbecco's modified eagle medium (cDMEM) containing 5 μg/mL Brefeldin A at 37°C in 5% CO_2_ for 4 h in the presence or absence of PMA and ionomycin. Cells were stained for cell surface markers and followed by intracellular staining. Intracellular staining was performed by using the BD Biosciences buffer system according to the manufacturer's instructions.

### Quantitation of CXCL16 in Lung Homogenates

The left side of the lung from each mouse was homogenized in 500 μl of T-PER™ tissue protein extraction reagent (ThermoFisher Scientific) containing proteinase inhibitor using a FastPrep-24™ 5G Instrument (MP Biomedicals). The lung homogenates were assayed using the mouse CXCL16 ELISA kit (ThermoFisher Scientific) according to the manufacturer's instructions.

### Statistical Analyses

All experiments were performed and repeated at least two to three time to assess reproducibility using three to five mice per experimental group unless otherwise stated in the figure legend. Data are represented as mean ± SEM and data were analyzed via one-way ANOVA followed by the Student-Newman-Keuls multiple stepwise comparison (for experiments with >2 experimental groups). A *P* < 0.05 was considered a significant difference (^*^P < 0.05, ^**^P < 0.01, ^**^P < 0.001).

## Results

### Lung MAIT Cells Are Predominantly CXCR6^+^ During Pulmonary *F. tularensis* LVS Infection

**Figure 1:**
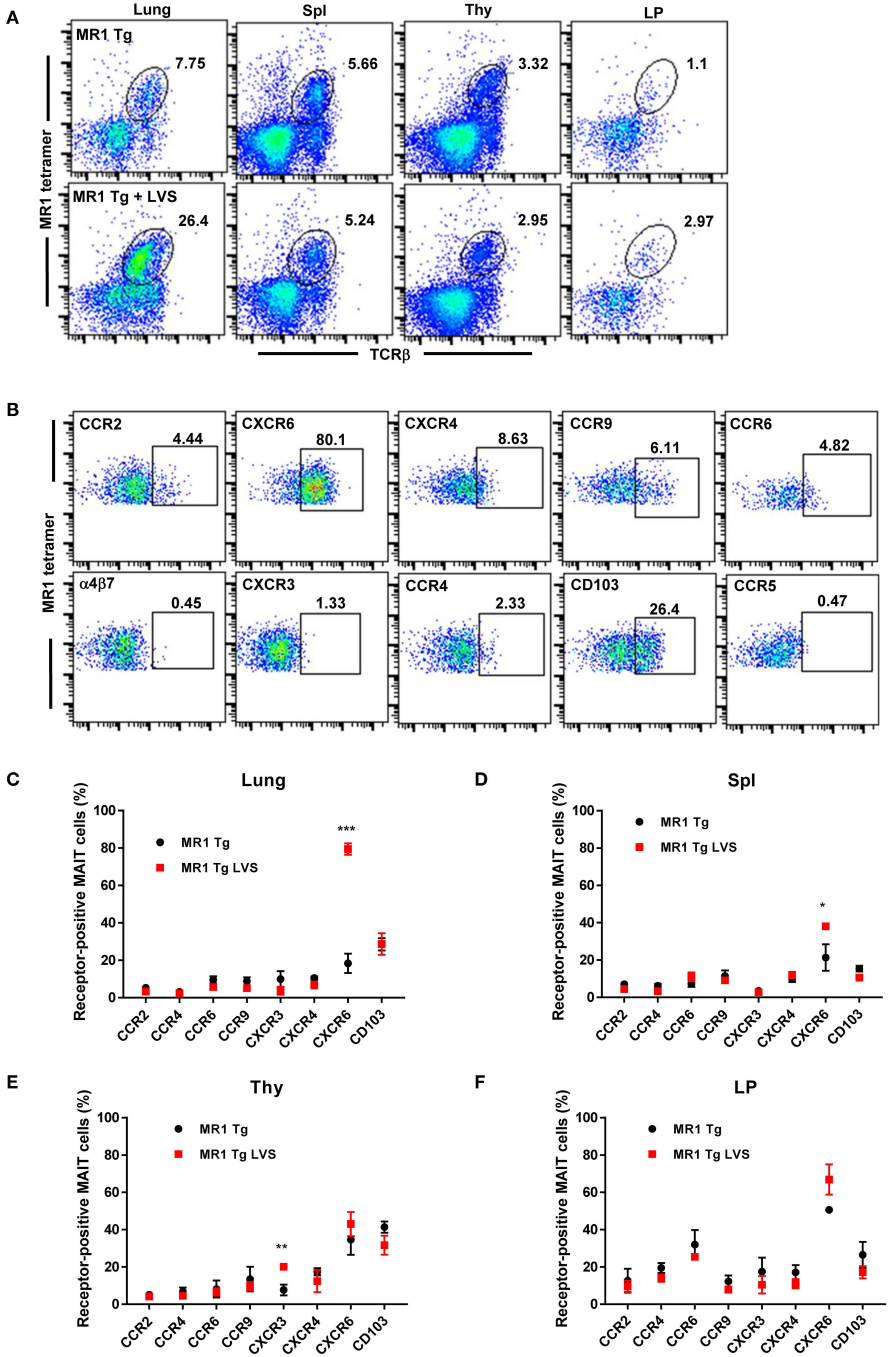
MAIT cells in the lungs of Vα19iTg mice predominantly express the chemokine receptor CXCR6 during *F. tularensis* LVS intranasal infection. **(A)** Representative flow cytometry dot plots of MAIT cells in the lungs, spleen (Spl), thymus (Thy), and intestinal lamina propria (LP) of naïve Vα19iTg mice (upper panel) and LVS-infected Vα19iTg mice on day 14 after infection (lower panel). MAIT cells are gated on live 5-OP-RU MR1 tetramer^+^ TCRβ^+^ cells in the total cell population for each organ. **(B)** Representative flow cytometry dot plots depicting expression of different chemokine receptors on MAIT cells in LVS-infected Vα19iTg mouse lungs on day 14 after infection. Plots show live 5-OP-RU MR1 tetramer^+^ TCRβ^+^ cells. A graphical representation of the percentage of chemokine receptor-positive MAIT cells in the lungs **(C)**, spleen **(D)**, thymus **(E)**, and intestinal lamina propria **(F)** in naive and LVS-infected Vα19iTg mice on day 14 after infection (*P < 0.05; **P < 0.01; ***P < 0.001 as compared to naïve mice). Data are presented as the mean ± SEM (*n* = 4–5) and are representative of two independent experiments.

To identify important chemokine receptors that may be used by MAIT cells for homing during infection, we first sought to characterize MAIT cell surface expression of different chemokine receptors during steady state and infectious conditions. Previous studies have shown that MAIT cell numbers in naïve pathogen-free wild type (WT) C57BL/6 mice are exceptionally low, constituting <0.2% of cells in the lungs, spleen, and thymus (24). In order to reliably assess the expression of chemokine receptors by MAIT cells under steady state conditions, we utilized transgenic mice that exclusively express the canonical MAIT cell Vα19-Jα33 TCRα chain (Vα19iTg mice). The gating strategy used to identify MAIT cells is shown in Figure S1. As expected, naïve Vα19iTg mice had high levels of MAIT cells in their lungs (7.8%), spleen (5.7%), thymus (3.3%), and lamina propria (LP; 1.1%), as shown in Figure 1A. Fourteen days following primary sublethal *F. tularensis* LVS intranasal (IN) infection, Vα19iTg mice exhibited significant accumulation of MAIT cells as compared to their naïve counterparts in the lungs (24.1 ± 2.3% vs. 9.1 ± 0.7%, *P* < 0.01; 2.1 × 10^6^ ± 7.2 × 10^4^ vs. 3.1 x 10^5^ ± 5.9 × 10^3^ total MAIT cells/lung, *P* < 0.01), but no significant changes were observed in the spleen (4.8 ± 0.3% vs. 5.2 ± 0.3%), thymus (2.0 ± 0.5% vs. 3.0 ± 0.6%), and LP (3.2 ± 0.4% vs. 1.9 ± 0.5%).

We next examined the surface expression of a panel of chemokine receptors and integrins by MAIT cells found in the lungs of naïve and LVS-infected Vα19iTg mice (Figures 1B–F, controls are shown in Figure S2). Of the ten chemokine receptors examined, only CXCR6 exhibited a significant change in the level of cell surface expression on MAIT cells in the lungs 14 days after LVS IN infection. As shown in Figure 1C, the percentage of CXCR6-expressing MAIT cells in the lungs increased almost five-fold in infected Vα19iTg mice (79.4% ± 1.7) as compared to naïve Vα19iTg mice (18.4% ± 3.0). A small but significant increase in the levels of CXCR6-expressing MAIT cells was also observed in the spleen, but not the thymus and LP after infection (Figures 1D,F). We found that relatively few MAIT cells expressed CCR2, CCR4, CCR6, CXCR3, CXCR4, and CCR9 in the lungs (Figure 1C), spleen (Figure 1D), thymus (Figure 1E), and LP (Figure 1F) both before and after infection. Of those chemokine receptors, only a slight increase in the percentage of CXCR3-expressing MAIT cells was observed after infection, and this only occurred in the thymus (*P* < 0.05). In addition, the mucosal integrin CD103 was expressed by about 20% of MAIT cells in the lung, spleen, thymus, and LP, but these levels did not significantly change following LVS IN infection.

**Figure 2:**
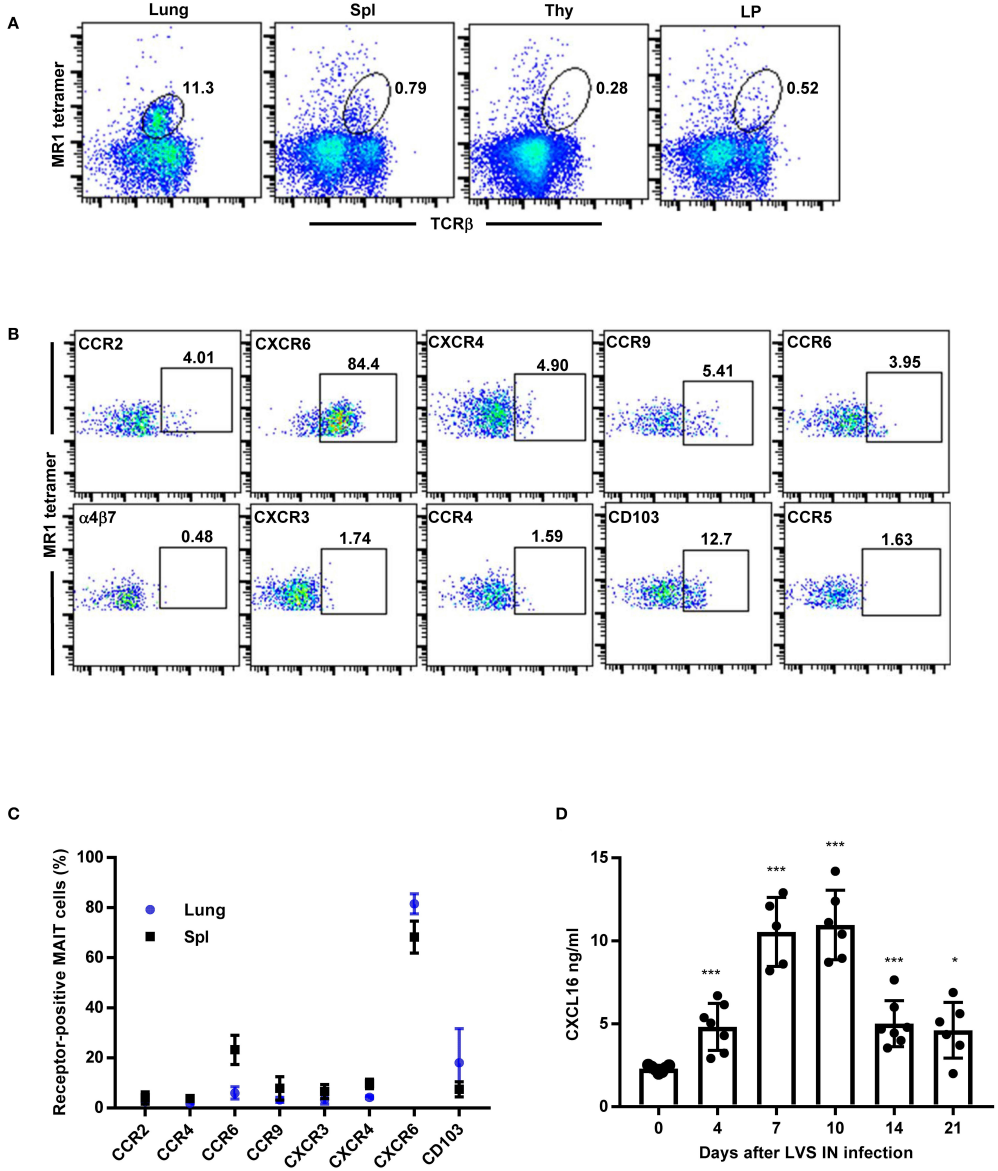
MAIT cells in the lungs of WT mice predominantly express the chemokine receptor CXCR6 during *F. tularensis* LVS intranasal infection. **(A)** Flow cytometry analysis of MAIT cells in the lungs, Spl (spleen), Thy (thymus), and LP (lamina propria) of LVS-infected WT mice on day 14 after LVS infection, showing reactivity to MR1-5-OP-RU tetramer in total lung cells. **(B)** Representative flow cytometry dot plots depicting expression of different chemokine receptors on MAIT cells in LVS-infected WT mice lungs (day 14 after infection). Plots show live 5-OP-RU MR1 tetramer^+^ TCRβ^+^ cells. **(C)** A graphical representation of the percentage of MAIT cells positive for the indicated chemokine receptors in the lungs and spleens (Spl) of LVS-infected WT mice on day 14 after infection. **(D)** The levels of CXCL16 in lung homogenates of naïve and LVS-infected WT mice at the indicated time points (*P < 0.05; ***P < 0.001 as compared to naïve Day 0 mice). Data are presented as the mean ± SEM (*n* = 5–6) and are representative of two independent experiments.

We next sought to determine whether MAIT cells exhibit a similar pattern of chemokine receptor expression in WT C57BL/6 mice during *F. tularensis* LVS IN infection. Fourteen days after LVS IN infection, large numbers of MAIT cells were observed in the lungs (9.1% ± 2.0), while relatively few were found in the spleen (0.9% ± 0.1), LP (0.5% ± 0.1), and thymus (0.2% ± 0.05; Figure 2A). Because such low numbers of MAIT cells were present in the LP and thymus, we did not examine chemokine receptor expression in these tissues. Consistent with our observations in LVS-infected Vα19iTg mice, MAIT cells in the lungs of infected WT mice were predominantly CXCR6^+^ (~80%; Figures 2B,C; controls are shown in Figure S2). In the spleen, ~60% of MAIT cells expressed CXCR6 and 20% expressed CCR6 (Figure 2C). Thus, a large proportion of MAIT cells in the lungs express the chemokine receptor CXCR6 during pulmonary LVS infection, suggesting that this receptor may participate in MAIT cell trafficking to the lungs.

### The CXCR6 Ligand CXCL16 Is Up-Regulated in the Lungs During Pulmonary *F. tularensis* LVS Infection

The only known ligand for CXCR6 is CXCL16, which was previously found to be expressed at high levels in the liver and lungs of naïve mice (32–34). Since the vast majority of MAIT cells responding to LVS pulmonary infection in the lungs were CXCR6^+^, we next investigated whether CXCL16 is elevated in the lungs during pulmonary LVS infection. To this end, we gave WT mice a sublethal LVS IN infection and CXCL16 levels were assessed in the lungs over time. As shown in Figure 2D, approximately 2 ng/ml of CXCL16 was detected in in the lungs of naïve WT mice. The levels of CXCL16 significantly increased as early as 4 days after LVS IN infection and peaked 7–10 days post-infection (an approximately five-fold increase as compared to naïve mice on day 10; *P* < 0.001). By 21 days after LVS infection, at a time when bacterial CFUs were fully cleared, the CXCL16 levels had diminished but not fully returned to that of naïve mice (*P* < 0.05). Thus, CXCL16 is significantly up-regulated in the lungs during LVS pulmonary infection and may serve as a mechanism to recruit CXCR6-expressing MAIT cells.

### CXCR6 Is Not Required for MAIT Cell Accumulation in the Airway Lumen and Lung Parenchyma During Pulmonary *F. tularensis* LVS Infection

**Figure 3:**
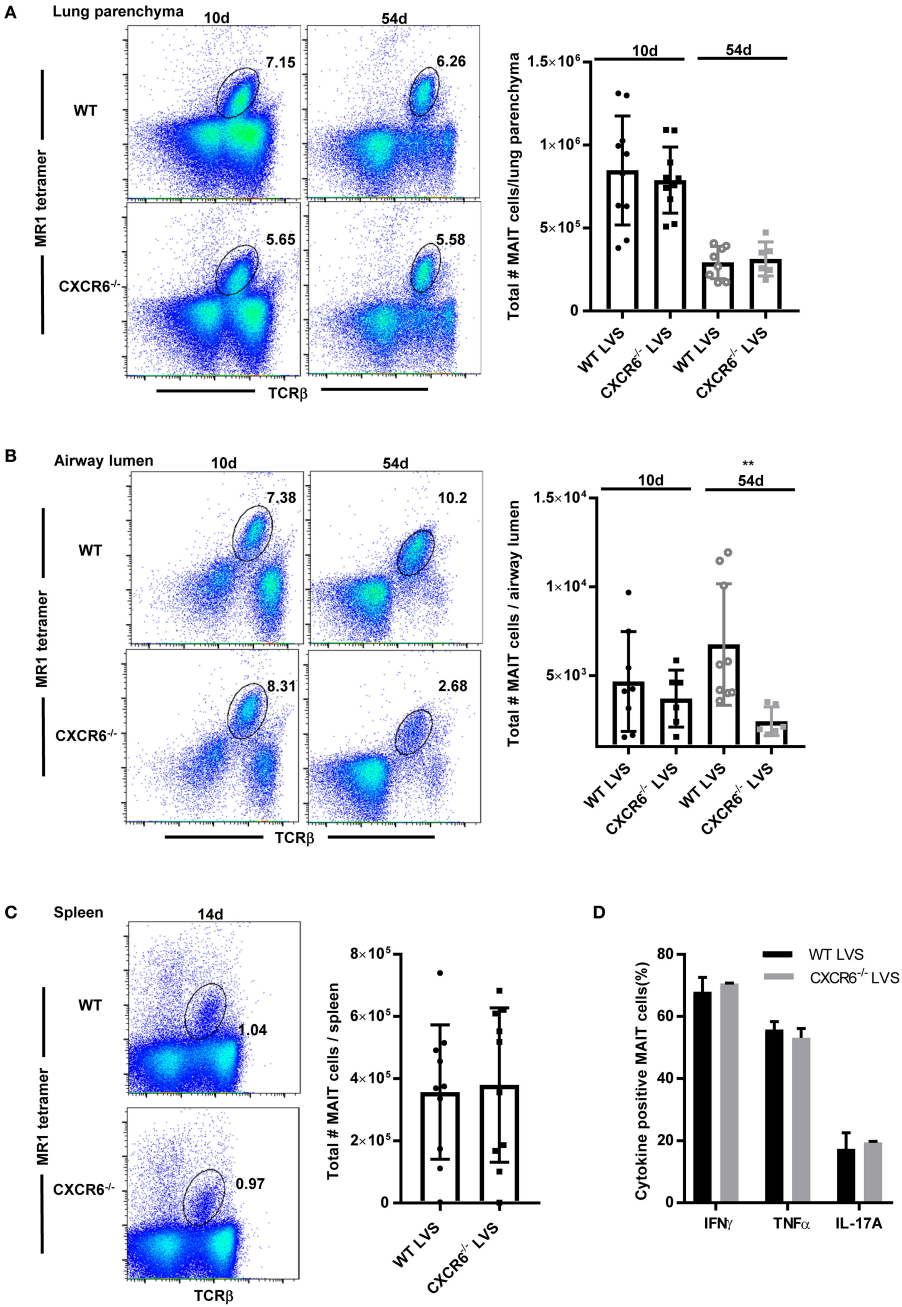
CXCR6 is not required for MAIT cell accumulation in the lung parenchyma or spleen during *F. tularensis* LVS intranasal infection but contributes to long-term retention in the airway lumen (BAL). **(A)** Representative flow cytometry dot plots showing the percentage of MAIT cells in the lung parenchyma of WT and CXCR6^−/−^ mice on days 10 and 54 after LVS IN infection. MAIT cells are gated on live MR1-5-OP-RU tetramer^+^ TCRβ^+^ cells in the lungs. A graphical representation of the number of MAIT cells in the lung parenchyma is shown. **(B)** Representative flow cytometry dot plots of the percentage of MAIT cells in the airway lumen (BAL) of WT and CXCR6^−/−^ mice on days 10 and 54 after LVS IN infection. MAIT cells are gated on live MR1-5-OP-RU tetramer^+^ TCRβ^+^ cells in the lungs. A graphical representation of the number of MAIT cells in the airway lumen is shown (**P < 0.01 as compared to WT mice). **(C)** Representative flow cytometry dot plots of the percentage of MAIT cells in the spleen of WT and CXCR6^−/−^ mice on day 14 after LVS IN infection. MAIT cells are gated on live MR1-5-OP-RU tetramer^+^ TCRβ^+^ cells in the spleen. A graphical representation of the number of MAIT cells in the spleen is shown. **(D)** Lungs harvested from LVS-infected WT and CXCR6^−/−^ mice on day 14 after infection were stimulated *in vitro* in the presence of PMA and ionomycin. A graphical representation of the percentage of MAIT cells positive for IFN-γ, TNF, and IL-17A is shown. Data are presented as the mean ± SEM (*n* = 5–7) and is representative of at least two independent experiments.

To further elucidate the role of CXCR6 in MAIT cell localization and function during infection, we compared MAIT cell accumulation in the lungs of CXCR6 knock out mice (CXCR6^−/−^) and WT mice after LVS IN infection. We previously found that MAIT cell numbers increased substantially after the first week of LVS pulmonary infection (days 8–9), reached a peak during the clearance phase of infection (days 10–14), and declined slowly thereafter but remained a notable presence in the lungs for a long time (>56 days) (9).Thus we examined MAIT cell accumulation in the lungs of CXCR6^−/−^and WT mice on days 10 and 54 after LVS IN infection. In addition, since recent research reported that the CXCR6/CXCL16 signaling axis regulated localization of tissue resident memory CD103^+^ CD69^+^ CD8^+^ T cells to the airway lumen (35), we examined MAIT cell partitioning in the lungs by assessing MAIT cell numbers in the lung parenchyma and airway lumen (BAL fluid). There were no differences in the total numbers of MAIT cells in the lung parenchyma (Figure 3A) and airway lumen (Figure 3B) on day 10. However, MAIT cell numbers in the airway lumen, but not the lung parenchyma, were significantly reduced in CXCR6^−/−^mice as compared to WT mice on day 54, long after clearance of the LVS infection (at approximately day 18). Of note, we observed no significant differences in LVS growth in the lungs between WT and CXCR6^−/−^ mice (Figure S2). MAIT cell numbers in the spleen were not significantly different between LVS-infected WT and CXCR6^−/−^ mice at a time when MAIT cell numbers peaked in that tissue (day 14) (Figure 3C). Overall, these data show that CXCR6 is not essential for MAIT cell accumulation in the lungs and spleen during LVS IN infection but has a significant role in maintaining MAIT cells in the airway lumen long after clearance of the infection.

Previous studies have shown that the loss of CXCR3 expression by MAIT cells in patients with end stage renal disease was coupled to increased expression of CCR6 and CXCR6 (36). To assess the possibility that other chemokine receptors may have compensated for the loss of CXCR6 in the CXCR6^−/−^ mice, we examined the cell surface expression of a panel of chemokine receptors on MAIT cells present in the lungs of infected WT mice and CXCR6^−/−^ mice. No significant changes were observed in the proportion of MAIT cells expressing the chemokine receptors CCR2, CCR4, CCR5, CCR6, CCR9, CXCR3, CXCR4, and α4β7 in LVS-infected CXCR6^−/−^ mice as compared to WT mice (data not shown). Thus, our evidence suggests that MAIT cell accumulation in the lungs of CXCR6^−/−^ mice is not the result of compensation via any of the aforementioned chemokine receptors.

Although we were unable to detect a difference in the number of MAIT cells present in the lungs of WT and CXCR6^−/−^ mice after LVS IN infection, it was possible that the MAIT cells accumulating in the lungs of the CXCR6^−/−^ mice had functional deficits. To this end, we compared cytokine production by MAIT cells in the lungs of LVS-infected WT and CXCR6^−/−^ mice. No significant differences were observed in the production of IL-17, IFN-γ, or TNF by MAIT cells obtained from the lungs of LVS-infected WT and CXCR6^−/−^ mice (data not shown). Similarly, no differences in cytokine production were detected by MAIT cells obtained from the same mice and further stimulated *ex vivo* with PMA/ionomycin (Figure 3D). Thus, MAIT cells from CXCR6^−/−^ mice do not have obvious deficits in IL-17, IFN-γ or TNF production.

### The Majority of MAIT Cells Proliferate in the Lungs During Pulmonary *F. tularensis* LVS Infection

**Figure 4:**
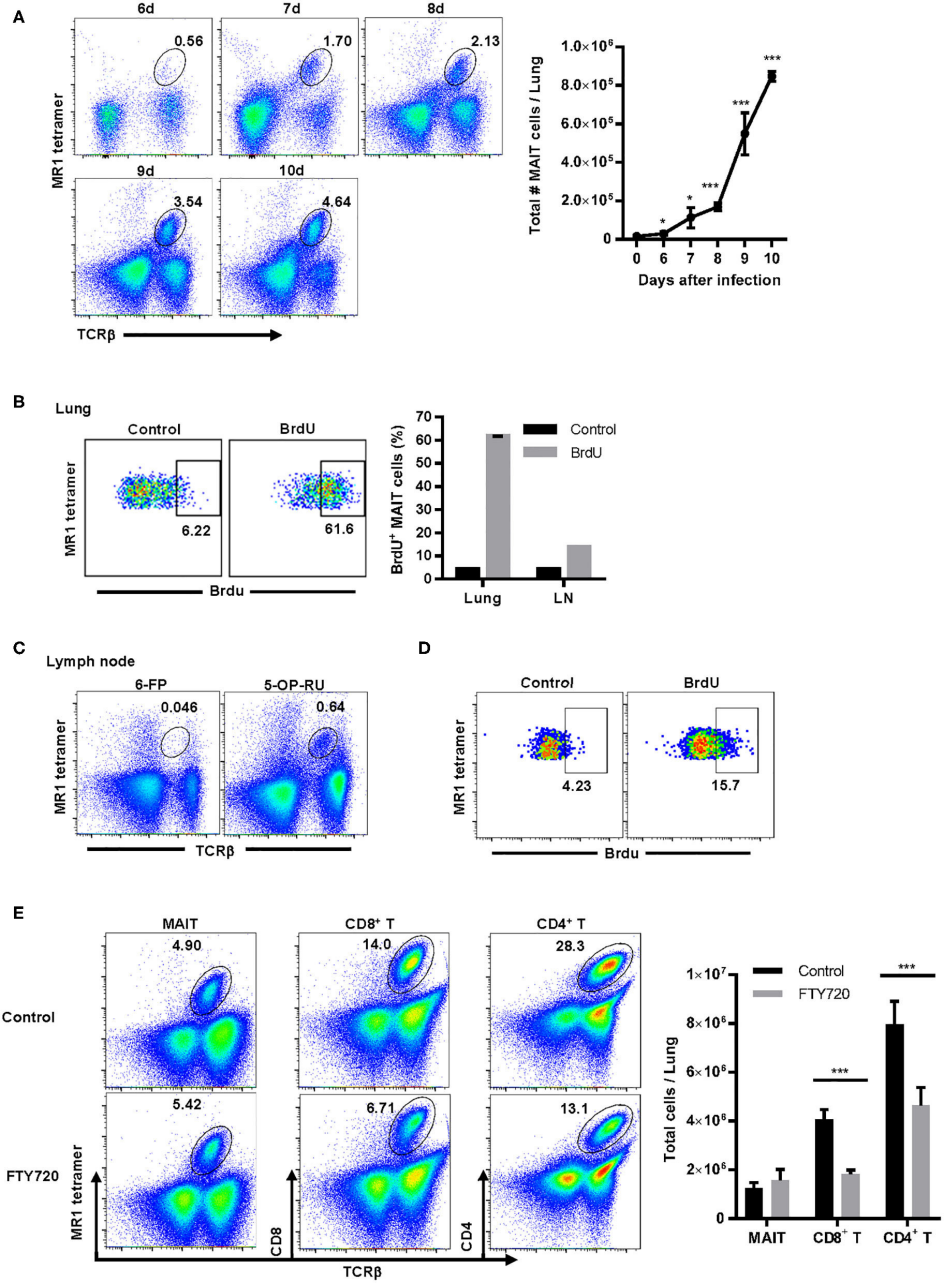
MAIT cells are not recruited from secondary lymphoid organs and largely proliferate *in situ* in the lungs during *F. tularensis* LVS pulmonary infection. **(A)** Representative flow cytometry dot plots of MAIT cells (gated on live 5-OP-RU MR1 tetramer^+^ TCRβ^+^ cells) in the lungs of WT mice on days 6–10 after LVS IN infection. A graphical representation of the total number of MAIT cells in the lungs is shown (*P < 0.05; ***P < 0.001 as compared to naïve mice). **(B)** Representative flow cytometry dot plots of MAIT cell incorporation of BrdU in the lungs of WT mice on day 10 after LVS IN infection. Mice were administered BrdU IN daily on days 6–9 after infection. Control staining was performed using an isotype control Ab on mice that received BrdU. MAIT cells were gated on live 5-OP-RU MR1 tetramer^+^ TCRβ^+^ cells. This is accompanied by a plot depicting the percentage of MAIT cells in the lungs and draining lymph nodes (LN) that were BrdU^+^ following staining with anti-BrdU Ab (BrdU) or isotype control Ab (Control). **(C)** Representative flow cytometry dot plots of MAIT cells in the cervical and mediastinal lymph nodes of WT mice on day 10 after LVS IN infection. Plots show staining with the negative control MR1 tetramer (6-FP) and the MAIT cell-reactive MR1 tetramer (5-OP-RU) in total lymph node cells. **(D)** Representative flow cytometry dot plots of MAIT cell incorporation of BrdU in the lymph nodes on day 10 after LVS IN infection. These dot plots show BrdU uptake by MAIT cells found in the lymph nodes of mice shown in **(B)** above. **(E)** Representative flow cytometry dot plots of MAIT cells, CD4^+^ T cells, and CD8^+^ T cells in the lungs of WT control mice and mice treated with the sphingosine-1-phosphate agonist FTY720 on days 2–9 after LVS IN infection. The lungs were harvested on day 10 after infection for flow cytometry analysis. The accompanying panel is a graphical representation of this data (***P < 0.001 as compared to control). Data are presented as the mean ± SEM (*n* = 3) and are representative of two independent experiments.

Since we were unable to identify any chemokine receptors that were likely to promote MAIT cell recruitment to the lungs during LVS infection, we next determined whether MAIT cells proliferated in the lungs during pulmonary LVS infection. As shown in Figure 4A, MAIT cell numbers in the lungs increased significantly starting on day 6 after LVS infection as compared to naïve mice. To determine whether this increase was the result of MAIT cell recruitment to the site of infection or *in situ* proliferation in the lungs, MAIT cells present in the lungs were given the opportunity to incorporate bromodeoxyuridine (BrdU) delivered via the IN route. BrdU is a synthetic nucleoside analog of thymidine that is only incorporated into actively replicating cells. BrdU was administered daily days 6–9 after infection, and BrdU^+^ MAIT cells were assessed in the lungs on day 10. Approximately 60% of MAIT cells were BrdU^+^ on day 10 after infection (Figure 4B), indicating that the majority of these cells had proliferated in the preceding 4 days.

Since previous studies showed that some of the BrdU delivered IN to mice travels to the lung-draining lymph nodes (37), it remained possible that some of the BrdU^+^ MAIT cells observed in the lungs had migrated from local lymph nodes. To address this possibility, we first examined the MAIT cell population in the mediastinal and cervical lymph nodes on day 10 after LVS IN infection. Although MAIT cells were a very small population in the lung-draining lymph nodes (Figure 4C), a significant proportion of these cells were BrdU^+^ at day 10 (Figure 4D). Therefore, to determine whether MAIT cells located in the lymph nodes traffic to the lungs during LVS IN infection, mice were administered the sphingosine-1-phosphate receptor agonist FTY720. FTY720 (fingolimod) is an FDA-approved drug for multiple sclerosis that inhibits lymphocyte egress from the thymus and secondary lymphoid organs (38). Mice were treated with FTY720 starting on day 2 after LVS IN infection, before MAIT cells increased significantly in the lungs, and daily administration was continued until we investigated the numbers of MAIT cells, CD4^+^ T cells, and CD8^+^ T cells in the lungs on day 10. As shown in Figure 4E, the numbers of CD4^+^ and CD8^+^ T cells were significantly reduced in the lungs of mice administered FTY720 as compared to control mice, indicating that a large proportion of these T cells trafficked to the lungs from secondary lymphoid organs. In contrast, the number of MAIT cells in the lungs of mice administered FTY720 was not significantly different from control mice, demonstrating that emigration from the lymph nodes and other secondary lymphoid organs does not substantially contribute to the MAIT cell population found in the lungs during infection. This evidence, coupled with the high levels of BrdU incorporated by lung MAIT cells after IN administration, suggest that the majority of MAIT cells in the lungs during pulmonary infection arise from *in situ* proliferation as opposed to trafficking from other sites.

### Intranasal Administration of CXCL16 and 5-OP-RU Induces MAIT Cell Accumulation in the Lungs Under Non-inflammatory Conditions

Previous studies have shown that intranasal administration of a TLR agonist (e.g., CpG, Pam_2_CSK_4_) and a MAIT cell activating antigen (e.g., 5-OP-RU) induced robust accumulation of MAIT cells in the lungs of naïve mice (10). In contrast, intranasal inoculation of a TLR agonist in combination with the MAIT cell inhibitory antigen Ac-6-FP failed to stimulate MAIT cell accumulation. Further, the TLR agonist and the MAIT cell antigen were both essential, as administration of either component individually was not sufficient to increase MAIT cell numbers in the lungs. TLR agonists are highly immunostimulatory and thus far it remains unclear which of the many cytokines and chemokines produced in response to these agents are sufficient to induce MAIT cell accumulation in combination with 5-OP-RU.

**Figure 5:**
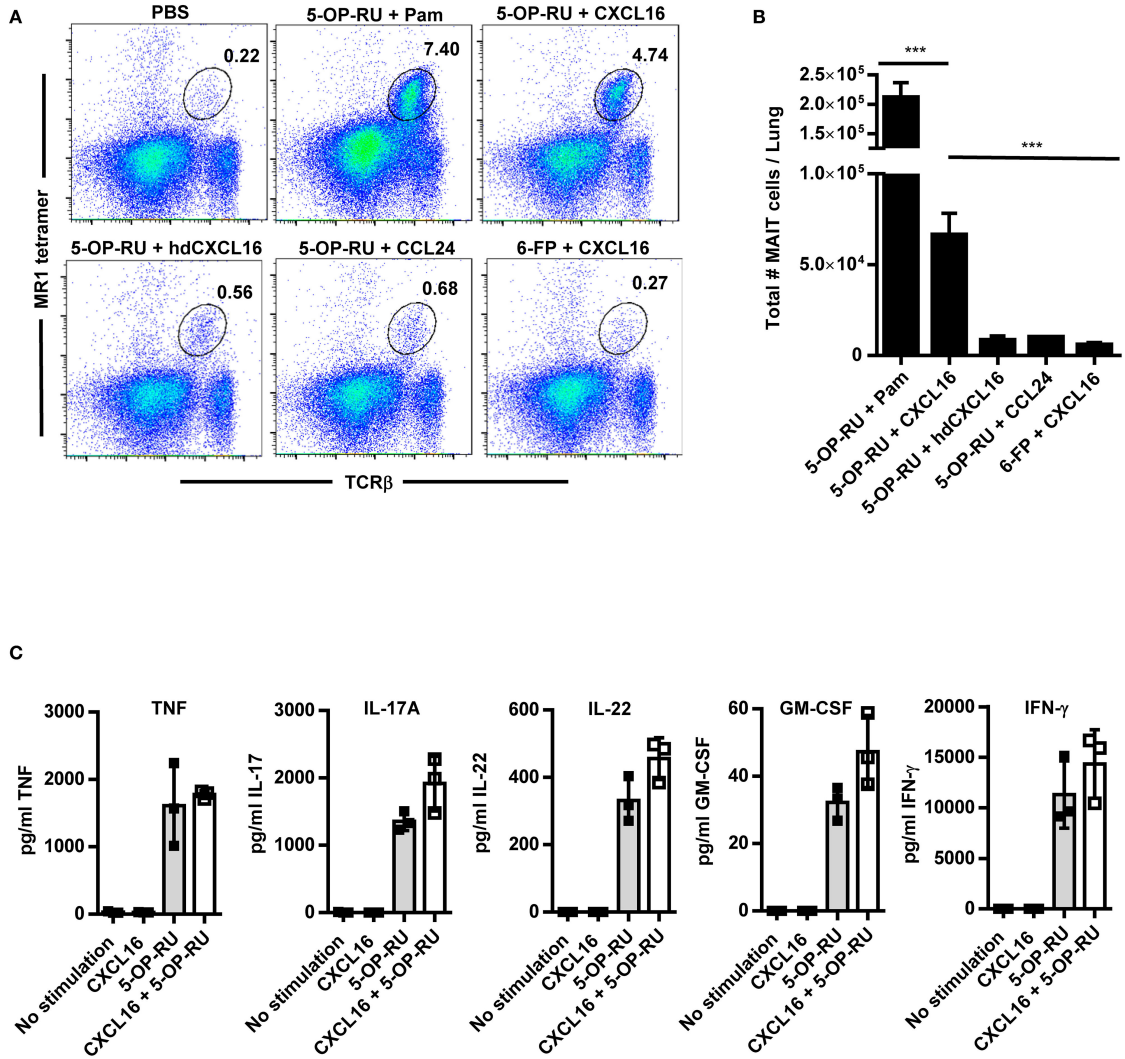
Intranasal instillation of CXCL16 and 5-OP-RU induces MAIT cell accumulation in the lungs in the absence of infection. Naïve WT mice were intranasally administered 5-OP-RU or control Ac-6-FP (6-FP) with one of the chemokines shown on days 1, 2, and 3. Mice given 5-OP-RU + Pam were intranasally administered 5-OP-RU and Pam on day 1, and 5-OP-RU alone on days 2 and 3. On day 7, the lungs were harvested for flow cytometry analysis. “PBS” mice received only PBS at the indicated time points. **(A)** Representative flow cytometry dot plots showing 5-OP-RU MR1 tetramer^+^ TCRβ^+^ MAIT cells in naïve WT mice treated as indicated. Total live singlet lung cells for individual mice are shown. **(B)** A graphical representation of the number of MAIT cells in the lungs of mice on day 7 (***P < 0.001). **(C)** Vα19iTgMR1^+/+^ transgenic murine MAIT cells were co-cultured with uninfected macrophages, recombinant CXCL16, and 5-OP-RU, and cytokine production was measured after 16 h. “No stimulation” = uninfected macrophages and MAIT cells. hd CXCL16 = heat-denatured CXCL16. Pam = Pam_2_CSK_4_. Data are presented as the mean ± SEM (*n* = 3) and is representative of three independent experiments.

Although our data show that CXCR6 is not required to increase MAIT cell numbers in the lungs during the peak of infection, this does not rule out a redundant role for the CXCR6/CXCL16 axis in MAIT cell accumulation. Since the majority of MAIT cells expressed CXCR6, we next sought to determine whether administration of CXCL16 might be sufficient to replace the aforementioned TLR agonists in the promotion of MAIT cell accumulation when combined with 5-OP-RU. To this end, recombinant CXCL16 and 5-OP-RU were administrated intranasally to naïve WT mice on day 1, and 5-OP-RU alone was administrated intranasally on days 2 and 3. MAIT cell numbers in the lungs on day 7 were then compared to several different control immunostimulatory combinations (Figures 5A,B). Consistent with previous research (10), the TLR2/6 agonist Pam_2_CSK_4_ + 5-OP-RU dramatically induced MAIT cell accumulation in the lungs (7.5 ± 0.1% of total lung cells). Significant accumulation of MAIT cells was also observed in mice that received CXCL16 + 5-OP-RU (3.7 ± 0.7%) on days 1, 2, and 3, but not in those that received CXCL16 + Ac-6-FP (0.4 ± 0.1%) or PBS (0.2 ± 0.02%) (Figures 5A,B). Recombinant CCL24 and heat denatured CXCL16 were used as controls to confirm the role of CXCL16 in the enrichment of MAIT cells. CCL24 binds the chemokine receptor CCR3, which is crucial for directing migration and priming of eosinophils and is unlikely to contribute to MAIT cell trafficking (39). When CCL24 + 5-OP-RU (0.6 ± 0.2%) or heat-denatured CXCL16 + 5-OP-RU (0.6 ± 0.1%) were administrated intranasally to naïve WT mice (Figures 5A,B), no significant MAIT cell accumulation was observed. Of note, mice treated with CXCL16 + 5-OP-RU did not exhibit significant differences in the numbers of conventional CD4^+^ and CD8^+^ T cells or inflammatory monocytes (Ly6C^hi^ CD11b^+^) as compared to mice given PBS (Figure S4). In contrast, mice administered CXCL16 + 5-OP-RU exhibited significantly more antigen presenting cells (CD11c^+^ MHCII^+^) as compared to PBS-treated mice, but not CXCL16 + Ac-6-FP-treated mice, indicating that this effect was not related to MAIT cell accumulation (Figure S4). Overall, these data demonstrate that CXCL16 in combination with 5-OP-RU is sufficient to induce MAIT cell accumulation *in vivo*.

To assess the ability of CXCL16 and CXCL16 + 5-OP-RU to stimulate MAIT cell cytokine production, purified transgenic murine MAIT cells were co-cultured with uninfected macrophages (for antigen presentation), recombinant CXCL16, and 5-OP-RU. As shown in Figure 5C, 5-OP-RU alone stimulated MAIT cells to produce multiple cytokines, including TNF, IL-17A, IL-22, GM-CSF, and IFN-γ. In contrast, CXCL16 alone failed to stimulate MAIT cell cytokine production. MAIT cells stimulated with the combination of CXCL16 + 5-OP-RU did not exhibit a significant increase in cytokine production as compared to those treated with 5-OP-RU alone. These data show that MAIT cells respond to CXCL16 + 5-OP-RU by producing critical cytokines, but that CXCL16 does not significantly augment the levels of MAIT cell effector cytokines produced in response to 5-OP-RU alone.

## Discussion

The chemokine receptor CXCR6 was originally described as the HIV and SIV co-receptor (40–42) and is expressed on NKT cells, a subset of activated T cells, and some NK cells (43–45). CXCR6 and its ligand CXCL16 have been shown to mediate homing of lymphocytes to non-lymphoid tissues as well as NK T cell homeostasis and activation (43, 45–48). Since several studies have shown that MAIT cells also express CXCR6 (21, 25), we were interested in determining its role in MAIT cell trafficking, cytokine production, and tissue localization.

Using the *F. tularensis* LVS pulmonary infection model, we found that the vast majority of MAIT cells in the lungs expressed CXCR6 during infection. Despite this finding, CXCR6 was not required for accumulation of MAIT cells in either the lung parenchyma or the airway lumen during LVS infection. Similarly, MAIT cell production of IFN-γ, TNF, and IL-17A was not significantly affected in CXCR6^−/−^ mice. Interestingly, however, CXCR6 had a significant role in maintaining MAIT cells in the airway lumen long after clearance of LVS infection. Importantly, CXCL16 is expressed in both soluble and membrane-bound forms (49), with the latter inducing adhesion of cells expressing CXCR6 (50). This is consistent with findings that suggest CXCL16 is weakly chemotactic (51) and instead has a role in controlling the localization of CXCR6^+^ cells within different compartments of the lung rather than directly recruiting them from the circulation (35). Indeed, CXCL16 is constitutively expressed by bronchial epithelial cells, which could facilitate retention of T lymphocytes in the lungs (35, 40, 52). Our own data identified CXCL16 as being up-regulated in the lungs during LVS infection and remained significantly elevated even after the bacterial CFUs had fully cleared. Overall, our data show that CXCR6 is not required for MAIT cell recruitment during infection but instead supports long-term retention of MAIT cells in the airway lumen.

Although our data does not rule out a role for chemokine receptors in MAIT cell recruitment, it raised the question of whether MAIT cells proliferate *in situ* in the lungs during pulmonary LVS infection. Of particular interest, Wang et al. found that MAIT cells in both the lungs and mediastinal lymph nodes proliferated during pulmonary *Legionella longbeacheae* infection (11). This study delivered BrdU via the intraperitoneal route and used a short labeling period (2 h) to avoid detecting cells that had proliferated elsewhere and migrated to the lungs. In our study, we delivered BrdU directly to the lungs by intranasal instillation and investigated the possibility that MAIT cells migrate from secondary lymphoid organs during infection. We found that the majority of MAIT cells in the lungs (~60%) and a smaller proportion in the lung-draining lymph nodes (~15%) incorporated BrdU during LVS pulmonary infection. Further, MAIT cell accumulation in the lungs was not impacted by treatment with the sphingosine-1-phosphate agonist FTY720, which inhibits lymphocyte trafficking from the thymus and secondary lymphoid organs (38). This was in sharp contrast to conventional CD4^+^ and CD8^+^ T cells, which exhibited impaired recruitment to the lungs after FTY720 treatment. Although FTY720 does not inhibit the migration of T cells found in non-lymphoid tissues and the blood, our combined data suggest that the majority of MAIT cells in the lungs arise from *in situ* proliferation during infection.

Several studies have demonstrated that MAIT cell accumulation in the lungs can be induced by intranasal instillation of a TLR agonist and a MAIT cell antigen in naïve mice (10, 53). Since TLR agonists stimulate production of multiple cytokines and chemokines, the precise agents that facilitate MAIT cell accumulation remain unknown. Further, TLR agonists are broadly inflammatory and may be detrimental in some situations where MAIT cells could be targeted as an immunotherapy. Here we found that intranasal administration of CXCL16 with 5-OP-RU was sufficient to increase MAIT cell numbers in the lungs of naïve mice, although it was not as effective as the TLR agonist Pam_2_CSK_4_. This is similar to the finding that intranasal delivery of the *Mycobacterium tuberculosis* antigen rec85A with CXCL16 induced enrichment of antigen-specific CXCR6^+^ T cells in the airways of previously immunized mice and provided significant protection against pulmonary *M. tuberculosis* infection (54). Although our data showed that CXCR6 was not essential for MAIT cell recruitment during LVS infection, this does not rule out a non-redundant role for the CCR6/CXCL16 axis in MAIT cell accumulation. However, it is particularly interesting to note that CXCL16 stimulated proliferation of Jurkat T cells and primary human CD4^+^ T cells *in vitro* (55). In this light, it is possible that CXCL16 in combination with 5-OP-RU stimulated MAIT cell proliferation rather than recruitment to the lungs. Overall, our data demonstrate that CXCL16 can partially replace TLR agonists in regimens used to induce MAIT cell accumulation, offering a more targeted method to achieve this goal.

In summary, here we found that the vast majority of MAIT cells were CXCR6^+^ during pulmonary *F. tularensis* LVS infection, but that CXCR6 was not required for MAIT cell accumulation in the lung parenchyma. However, CXCR6 contributed to long-term retention of MAIT cells in the airway lumen. Our data show that MAIT cells are not recruited from secondary lymphoid tissues and largely proliferate *in situ* in the lungs during infection. Importantly, we found that CXCL16 can partially substitute for TLRs in therapeutic regimens that induce MAIT cell accumulation via administration of 5-OP-RU. Overall, our data advance the understanding of the mechanisms that facilitate MAIT cell accumulation and retention in the tissues.

## Data Availability Statement

All datasets presented in this study are included in the article/Supplementary Material.

## Ethics Statement

The animal study was reviewed and approved by FDA White Oak Animal Care and Use Committee.

## Author Contributions

SC and HY designed the study and analyzed and interpreted data. HY and AY performed experiments. LL, JM, and DF provided reagents. SC, HY, LL, JM, and DF wrote the manuscript. All authors contributed to the article and approved the submitted version.

## Conflict of Interest

The authors declare that the research was conducted in the absence of any commercial or financial relationships that could be construed as a potential conflict of interest.
