# Supplementary material for: CXCL16 Stimulates Antigen-Induced MAIT Cell Accumulation but Trafficking During Lung Infection Is CXCR6-Independent
Source: Front Immunol. 2020 Aug 7;11:1773. doi: 10.3389/fimmu.2020.01773 (PMC7426740; doi:10.3389/fimmu.2020.01773)
Supplement: Supplementary file 1 [file Data_Sheet_1.PDF]

Figure S1

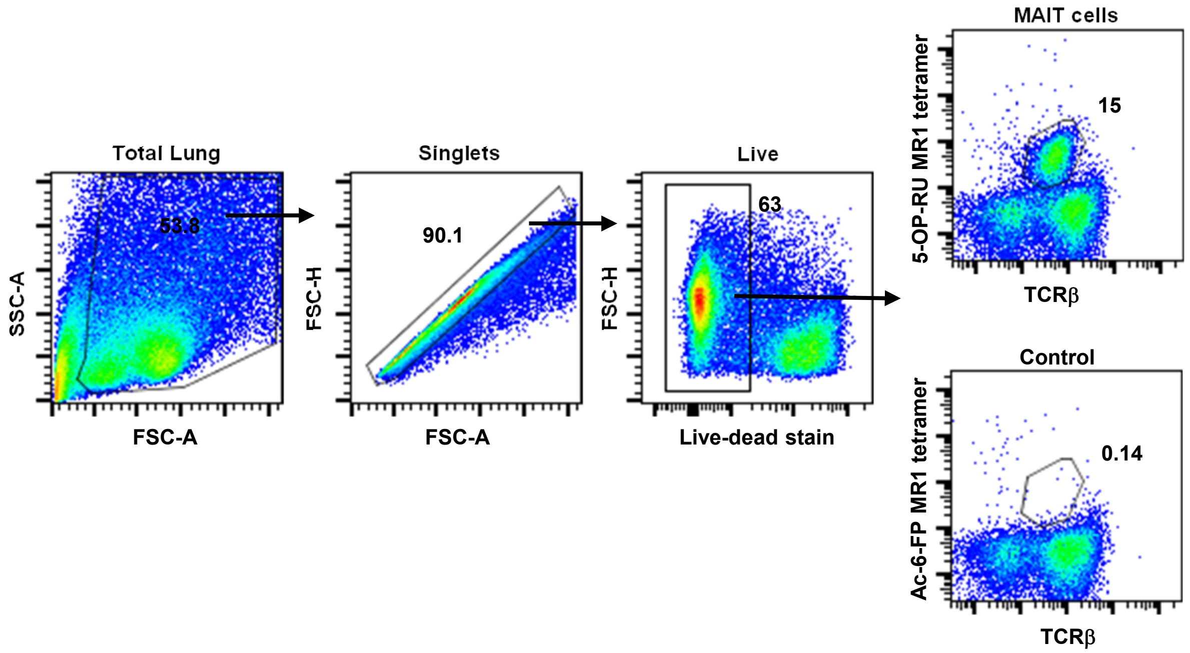

Figure S1. Representative MAIT cell gating strategy. Dot plots depict the gating strategy used to identify MAIT cells (total lung cells, singlets, live cells and 5-OP-RU MR1-tetramer^{+} TCRβ^{+} MAIT cells or control Ac-6-FP MR1 tetramer) in the lungs of a WT mouse 14 days after 10^{2} LVS intranasal infection.

Figure S2

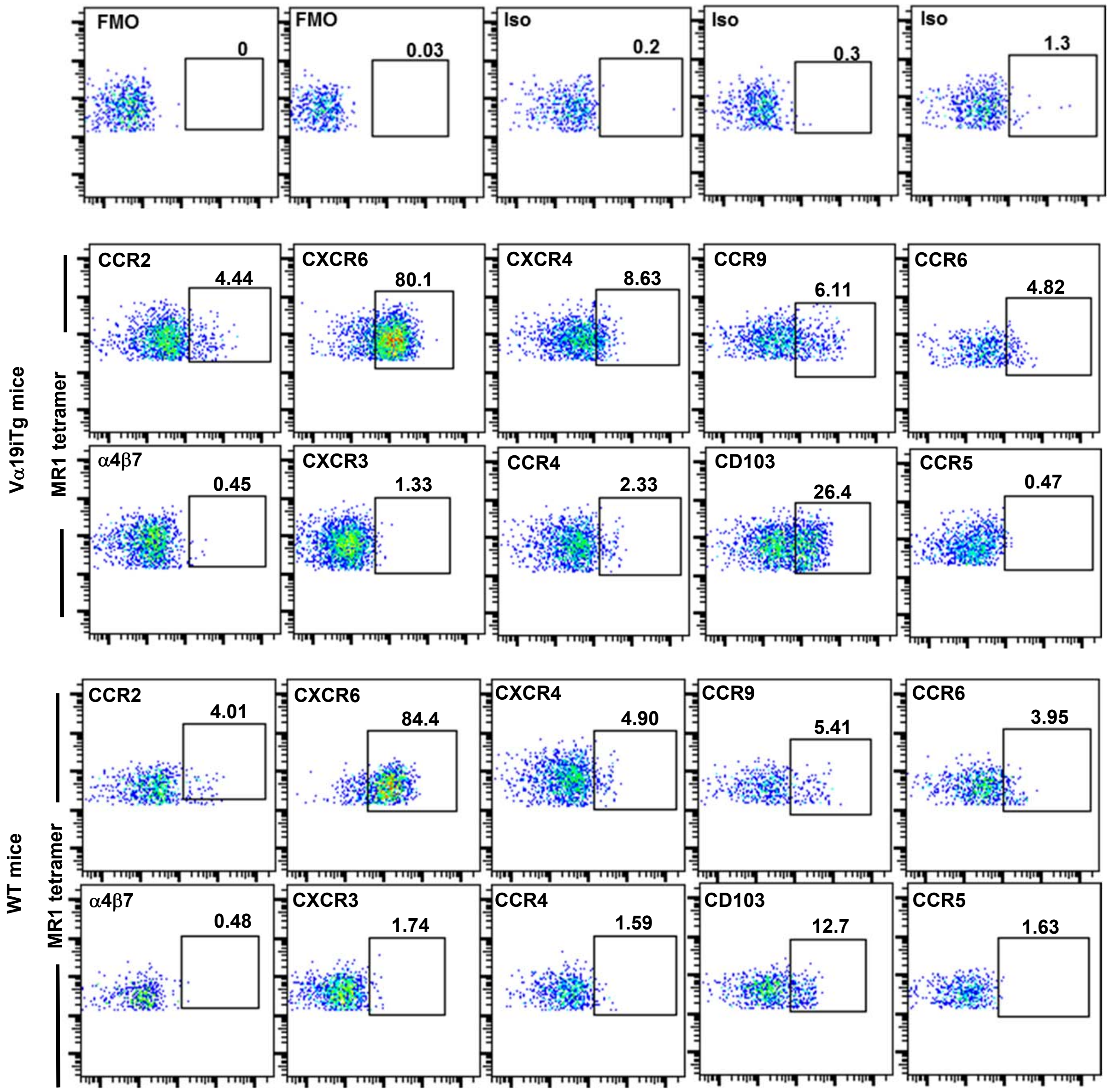

Figure S2. Control staining for MAIT cell chemokine receptor staining via flow cytometry. Flow cytometry dot plots are shown for the FMO and isotype controls (Iso) corresponding to the data shown in Figures 1B and 2B. The controls are shown alongside the relevant chemokine receptor staining dot plots for MAIT cells in LVS-infected Vα19iTg mouse lungs and LVS-infected C57BL/6 WT mouse lungs on day 14 after infection. All plots were first gated on 5-OP-RU MR1 tetramer$^+$ TCRβ$^+$ cells.

Figure S3

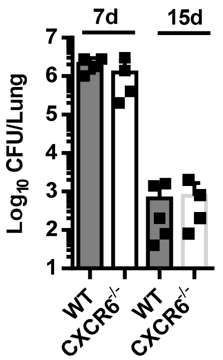

Figure S3. *F. tularensis* LVS growth in the lungs of WT and CXCR6$^{-/-}$ mice. LVS CFUs were enumerated in the lungs of WT and CXCR6$^{-/-}$ mice 7 and 15 days after sublethal 2×10^2 LVS IN infection. No significant differences were detected between the WT and CXCR6$^{-/-}$ mice (n = 4-5 mice).

Figure S4

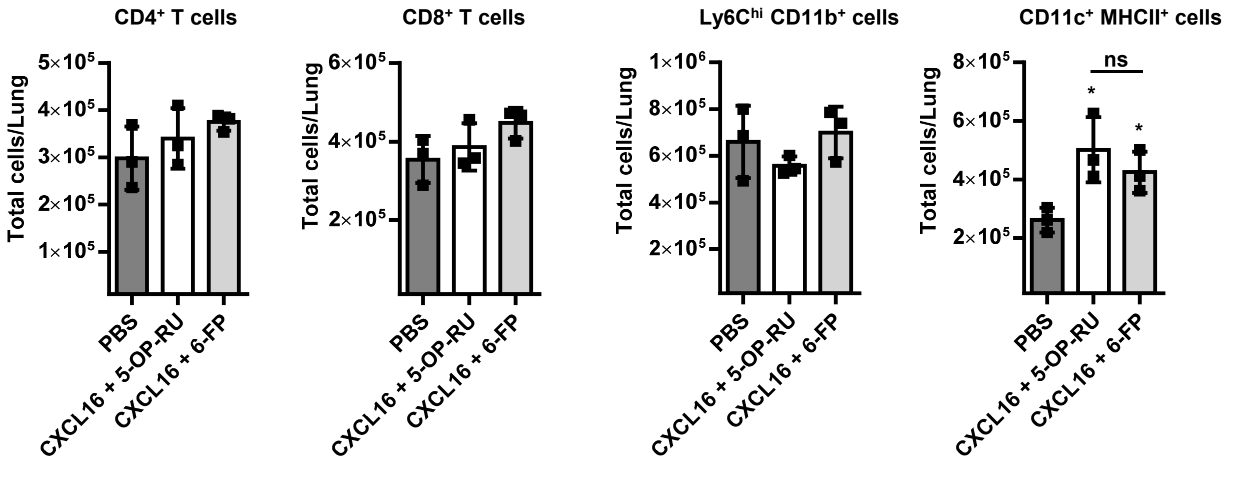

Figure S4. Effect of CXCL16 and 5-OP-RU administration on cellular recruitment to the lungs. Naïve WT mice were intranasally administered 5-OP-RU or control Ac-6-FP (6-FP) with recombinant CXCL16 on days 1, 2, and 3. Control mice were administered PBS. On day 7, the lungs were harvested for flow cytometry analysis. A graphical representation of the number of CD4$^+$ T cells, CD8$^+$ T cells, inflammatory monocytes (Ly6C^{hi} CD11b$^+$), and total antigen presenting cells (CD11c$^+$ MHCII$^+$) in the lungs of mice on day 7 (*, P<0.05 as compared to PBS mice). ns = not significant. Data are presented as the mean ± SEM (n = 3) and is representative of two independent experiments.
